# Implementing digital interventions in psychiatric outpatient units: a qualitative analysis of staff attitudes

**DOI:** 10.1186/1940-0640-10-S2-O6

**Published:** 2015-09-24

**Authors:** Anne H Berman, Ann-Sofie Bakshi, Kristina Sinadinovic, Christopher Sundström

**Affiliations:** 1 Karolinska Institute, Department of Clinical Neuroscience, Center for Psychiatric Research, Stockholm, Sweden; 2 Stockholm Center for Dependency Disorders, Stockholm, Sweden

## Background

Up to one-third of patients in psychiatry use alcohol or drugs at a level that is problematic. A national survey of outpatient psychiatric clinic directors and staff in Sweden was carried out on guidelines and practices regarding screening, brief intervention and referral to treatment (SBIRT) for their patients, showing that national recommendations to offer SBIRT for problematic substance use (PSU) are not systematically followed[1]. The objective of this research is to investigate whether implementation of digital interventions for PSU in psychiatry could be a way of increasing treatment access for patients and supporting staff in offering treatment.

## Material and methods

Clinic directors at seven psychiatric outpatient clinics in Stockholm, Sweden, were interviewed regarding their views on SBIRT for patients in psychiatry as well as the possibilities of implementing a digital stepped care model for offering SBIRT at their clinics. Interviews were transcribed verbatim and subjected to qualitative thematic content analysis.

## Results

PSU complicates correct diagnostic assessment and effective treatment intervention in psychiatry, and patients with PSU are generally referred to the addiction treatment clinic. However, patients neglect to attend the addiction clinic or do not complete treatment, due to the stigmatic nature of problematic substance because attending parallel treatments at two different clinics is taxing for patients. Interviewees were positive to the digital stepped care concept for psychiatry patients. Implementation would be contingent on easy use and minimal expenditure of staff time and resources. A barrier is that treating PSU is not perceived as part of psychiatry's mission and discipline.

## Conclusions

Implementing a digital stepped care concept for PSU within psychiatry could improve patient access to SBIRT and positively influence psychiatric treatment outcomes. Facilitating factors for implementation are user-friendly design and minimal time and resource requirements. A potential barrier is that staff do not perceive PSU treatment as part of their mission and area of competence.
